# Rare variants analysis using penalization methods for whole genome sequence data

**DOI:** 10.1186/s12859-015-0825-4

**Published:** 2015-12-04

**Authors:** Akram Yazdani, Azam Yazdani, Eric Boerwinkle

**Affiliations:** 1 Human Genetics Center, University of Texas Health Science Center at Houston, TX, USA; 2 Human Genome Sequencing Center, Baylor College of Medicine, Houston, TX USA

**Keywords:** Penalization, Linkage disequilibrium, Principal component, Rare variants, Sparsity

## Abstract

**Background:**

Availability of affordable and accessible whole genome sequencing for biomedical applications poses a number of statistical challenges and opportunities, particularly related to the analysis of rare variants and sparseness of the data. Although efforts have been devoted to address these challenges, the performance of statistical methods for rare variants analysis still needs further consideration.

**Result:**

We introduce a new approach that applies restricted principal component analysis with convex penalization and then selects the best predictors of a phenotype by a concave penalized regression model, while estimating the impact of each genomic region on the phenotype. Using simulated data, we show that the proposed method maintains good power for association testing while keeping the false discovery rate low under a verity of genetic architectures. Illustrative data analyses reveal encouraging result of this method in comparison with other commonly applied methods for rare variants analysis.

**Conclusion:**

By taking into account linkage disequilibrium and sparseness of the data, the proposed method improves power and controls the false discovery rate compared to other commonly applied methods for rare variant analyses.

## Background

Despite success in detecting associations of common variants with complex traits (www.genome.gov/gwastudies/), it has proven difficult to elucidate a comprehensive picture of the genetic architecture of risk factor and disease traits without considering the effects of both rare and common variants via whole exome or genome sequencing. Decreasing costs and increasing quality have made discovery and genotyping of rare variants, which refer to variants with minor allele frequency (MAF) less than 0.05, more accessible across a large proportion of the genome and in large sample sizes. As a result of rapid expansion of human populations, there are very large numbers of rare variants segregating and these rare variants are relatively recent in origin [1, 2]. Detecting genotype-phenotype associations and identifying novel loci having rare variant-phenotype associations are challenging since single-variant based statistical methods are inappropriate in this context due to the very large number of alleles and their low frequency. Furthermore, no or minimal effects of the majority of rare variants on a particular phenotype leads to a low signal-to-noise ratio and consequently underfitting with multiple-variant models. Hence, there is considerable interest in statistical methods that combine information across multiple variants, and thus reduce the cost of the large degrees of freedom in multivariate tests or adjustment for extensive multiple testing [3–9]. However simply combining information by pooling or collapsing does not take into account the direction of the variants’ effects on a phenotype and alternative methods have been proposed that address this limitation (see, e.g. [10–17]). Furthermore, inclusion of large numbers of correlated variants may lead to overestimation.

Transitioning from common variant analyses to rare variant analyses creates three challenges related to sparse data [18]. First, within an individual personal genome, the number of sites that differ from the reference genome is small relative to the total number of bases. Second, sequence data, unlike array-based genotype data, contain a large number of rare variants. In fact, about half of the variant alleles in a study sample are seen only one or two times [19]. And third, only a small subset of the variable sites is expected to influence a given trait of interest, and the rest is expected to be neutral. This study presents a statistical and computational method tailored for sparse data and how it can be applied to whole genome sequence data to promote novel gene and rare variant discovery. We introduce a new method called Convex-Concave Rare variant Selection (CCRS), which includes both convex and concave penalization. We leverage the fact that rare variants data have low intrinsic dimensionality and are sparse. Hence, we project the variants into a full rank space with new coordinates in order to enhance information in new predictors comparing with original variants. We obtain these new coordinates using principal component analysis that includes a convex penalty to incorporate sparsity assumption. The CCRS improves the performance of sparse principal component (SPC) based method [20] in the context of rare variants analysis by selecting the components based on their degree of association with a complex trait which is appropriate for rare variant analysis. To this end, we use a concave penalized regression model to select the most promising variants while estimating their effect simultaneously.

## Method

The CCRS method is applicable for all variants, but in this presentation, we focus on the analysis of rare variants because they pose special opportunities (i.e. large effects sizes) and challenges (i.e. sparsity). Assume we have detected and genotyped *m* rare variants **X**=(**x**_1_,…,**x**_*m*_) in a sample of *n* individuals having a quantitative trait **y**=(*y*_1_,…,*y*_*n*_) measured on each individual. In a typical whole exome or genome sequencing scenario, *m* is several orders of magnitude larger than *n*. To combat over-determination, the typical analysis considers a subset of the variables at a time defined by physical proximity (e.g. a window) instead of functional characteristic (e.g. an annotated gene or enhancer element) because the vast majority of the rare variants are in noncoding regions in the genome. Interpretation of the results requires adjusting for multiple comparisons using accepted experiment-wise error or false discovery rate methods. Assume for the *k*th subset of **X** denoted by $\mathbf {X}_{k}=\{\mathbf {x}_{\textit {jk}}\}_{j=1}^{p}$ where *p*<*n*, we have

(1) $$  \mathbf{y}=\alpha+\mathbf{X}_{k} \boldsymbol{\beta}_{k} + \mathbf{T}\boldsymbol\theta+ \boldsymbol\epsilon, \quad \boldsymbol\epsilon\sim N(0, \Sigma)  $$

where ***ε***=(*ε*_1_,…*ε*_*n*_) is an error vector, *Σ* is an *n*×*n* diagonal matrix; *α* is the overall mean; **T** is an *n*×*q* covariate matrix, which includes non-genetic predictors such as age, sex and race; ***β***_*k*_ and ***θ*** are *p*-vector of genetic effects and *q*-vector of non-genetic effects, respectively. Although model (1) does not face the *n*≪*p* problem, the data lie in a lower-dimensional subspace due to dependency among rare variants [21, 22] (i.e. linkage disequilibrium, LD) and coefficients are sparse because a large proportion of variants have small or no effects on the phenotype(s) of interest. Here, we introduce a new approach for rare variants analysis to address these two issues; LD and sparsity.

### The CCRS approach

In rare variant analyses, the design matrix is more likely to be singular because of the LD structure in the population [21, 22]. In addition due to low allele frequencies, there is little information about the association of each individual variant with a phenotype. Hence, applying a penalized regression model might not lead to identifying the true set of variants or genomic regions with nonzero effects. To bypass this difficulty, we project the genotype data into full rank space in order to reparameterize the regression model. Principal component analysis (PCA) is an appropriate tool for addressing collinearity and utilizes the low rank structure of the covariance matrix. One drawback of PCA is its lack of straight forward interpretability. However, in rare variant analyses each single variant is uninformative and there is a need to aggregate information in a region in order to identify association with the trait of interest. An issue of concern when applying PCA in the context of rare variants is that PCA may lead to new coordinates that include many non-influential variants due to sparseness. Accounting for such sparsity facilities identification of phenotype-influencing factors in each of the coordinates and also improves interpretability of the result because of the sparse loading matrix [23–25]. To accomplish this, we obtain a full rank approximation to the matrix **X** as

$$\mathbf{X}\approx \mathbf{U}_{n\times r} \mathbf{D}_{r\times r} \mathbf{V}_{r\times p} $$

by imposing constraints on the columns of *V* and *U* similar to [20],

(2) $$\begin{array}{*{20}l} &\parallel \mathbf{v}_{j} {\parallel_{2}^{2}} \leq 1 \quad \text{and} \quad \parallel \mathbf{v}_{j}\parallel_{1}\leq c \end{array} $$

(3) $$\begin{array}{*{20}l}  &\parallel \mathbf{u}_{j} {\parallel_{2}^{2}} \leq 1 \quad \text{and} \quad \mathbf{u}_{1}\perp \ldots\perp \mathbf{u}_{r} \end{array} $$

where *r* is the rank of **X**, which is smaller than min(*n,p*); ∥.∥_*a*_ denotes *L*_*a*_ norm; and $D=\{ d_{j} \}_{j=1}^{r}$ is a diagonal matrix of eigenvalues of the matrix **X** such that *d*_1_≥*d*_2_≥…≥*d*_*r*_; **v**_*j*_ and **u**_*j*_ are the *j*th columns of **V** and **U** respectively. The *L*_1_ norm penalization is equivalent to $\sum _{r} \mid v_{\textit {ij}}\mid $, where *v*_*ij*_ is *ij*th entry of **V**, provides sparse principal components, **U** **D**. This is an optimization problem equivalent to maximizing $\mathbf {u}_{j}^{T} \mathbf {X} \mathbf {v}_{j}$ respect to **u**_*j*_ and **v**_*j*_ under constraint (2) and (3). This biconvex problem can be readily solved [26]. Therefore, we first fix **u**_*j*_ and obtain **v**_*j*_ when *c* is in the set of feasible solution $ \{ c \mid 1< c < \sqrt {p} \}$. We then obtain optimum solution of **u**_*j*_ when $ \parallel \mathbf {u}_{j} {\parallel _{2}^{2}} \leq 1$ and for *j*>1, **u**_*j*_⊥**u**_1_,**u**_2_,…,**u**_*j*−1_. The optimal value of *c*, which determines the level of sparsity, can be obtained through a cross validation approach [27, 28].

By projecting data into a lower dimensional space, we reduce the number of predictors in the model to the rank of the design matrix, which increases the degree of freedom for hypothesis test and aggregates information into fewer predictor variables which helps alleviate one aspect of the low allele frequency challenge. These two features improve the power of identifying promising genetic regions influencing a phenotype of interest (see below).

In this context, it is not appropriate to select only the first few principal components as is usual in many applications, but rather we select the PCs based on their degree of association with the phenotype. To simultaneously measure the genotype-phenotype association and carry out variable selection, we consider a linear regression model including a concave penalization with loss function

(4) $$ \frac{1}{2}\parallel\mathbf{y}-\mathbf{Z}\boldsymbol\gamma - \mathbf{T}{\boldsymbol\theta\parallel_{2}^{2}} + \nu \parallel \boldsymbol\gamma \parallel_{1}^{\kappa}  $$

where **Z**=**U** **D** indicates a matrix of computed PCs with corresponding effect size ***γ***, *κ*∈(0,1) and regularization parameter $\nu \in \mathbb {R}^{+}$. Without loss of generality, hereafter, we assume the overall mean is zero.

This model is a form of Bridge regression and naturally yields sparse estimate for ***γ***, in the sense that some of components of ***γ***_(*κ*,*ν*)_, may be explicitly shrunk to zero [29, 30]. The choice of *κ*<1 leads to nonconcave minimization problems (see, e.g., [30–32]) and provides a much sparser solution than the well-known penalized regression, lasso, with *κ*=1 [33].

## Result

### A simulation study

**Fig. 1:**
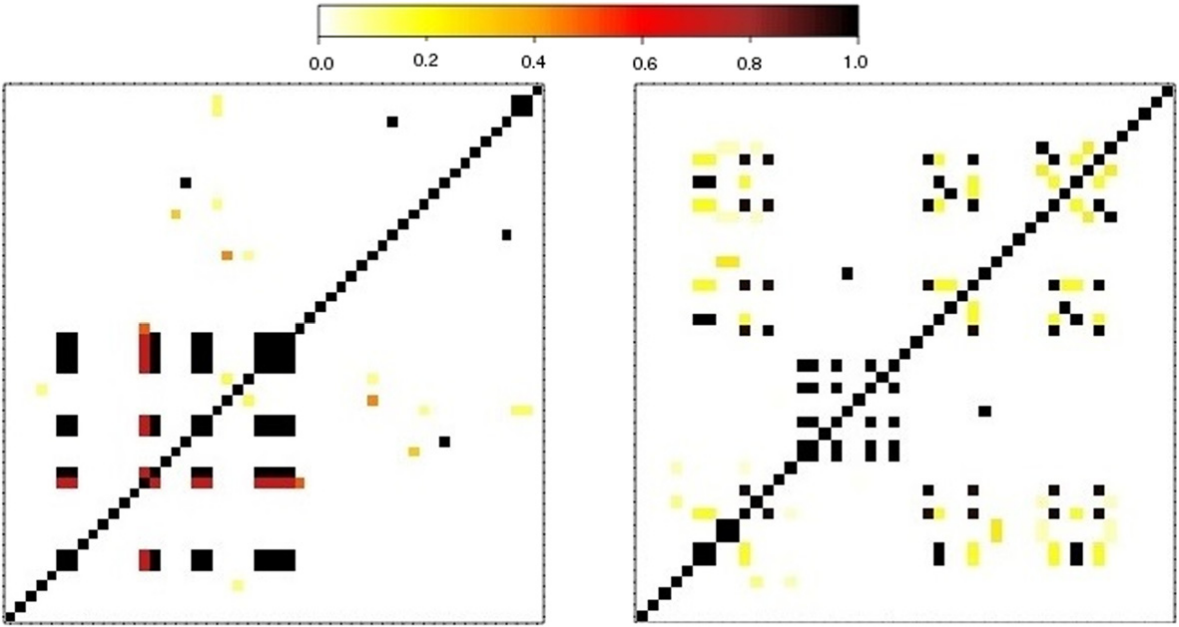
LD among variants with MAF <.05 in two different regions of the genome

To evaluate the performance of the CCRS method, we randomly identified 1000 regions from a real whole genome sequence data set available from phs000668 study in *dbGAP* (http://www.ncbi.nlm.nih.gov). Each region includes 50 variants (50,000 rare variants total) sequenced for 1456 individuals. Based on our experience, we have found that 50 variants are appropriate to capture the LD structure. As an example, Fig. 1 represents this LD structure for two regions of the genome.

We considered six different phenotypic effect scenarios (Table 1). We first randomly split the set of regions into two subsets to be influential regions and noninfluential regions. We then randomly selected 10 *%* of variants in each influential region to be causal variants with effect size +1 for *Model-1* and *Model-3* and with effect size ±1 for *Model-2* and *Model-4*. In *Model-5* and *Model-6*, the number of causal variants in a region is increased to 20 *%* of the total variants with different effect sizes randomly selected from U(0.5,1) and {U(−1,−0.5),U(0.5,1)}, respectively, where U denotes uniform distribution. Hence, we considered models with the same and also different effect directions.

**Table 1:** Six genotype effect scenarios considered in simulation studies

*Model-1:*	10 *%* of variants in influential regions are causal with effect size +1, while each one is correlated with some neutral variants in their region.
*Model-2:*	10 *%* of variants in influential regions are causal with effect size ±1, while each one is correlated with some neutral variants in their region.
*Model-3*:	10 *%* of variants in influential regions are causal with effect size +1, while they are uncorrelated with other variants in their region.
*Model-4:*	10 *%* of variants in influential regions are causal with effect size ±1, while they are uncorrelated with other variants in their region.
*Model-5:*	20 *%* of variants in influential regions are causal with effect size selected from *U*(0.5,1), while 10 *%* are correlated and 10 *%* are uncorrelated with other causal and neutral variants in their region.
*Model-6:*	20 *%* of variants in influential regions are causal with effect size selected from *U*(−1,−0.5) and *U*(0.5,1) while 10 *%* are correlated and 10 *%* are uncorrelated with other causal and neutral variants in their region.

To obtain a better understanding about the effect of LD on the result of the analysis, we selected variants based on their correlations. In *Model-1* and *Model-2*, the causal variants are correlated with some neutral variants in their regions but in *Model-3*, *Model-4* they are uncorrelated. For *Model-5* and *Model-6*, both correlated and uncorrelated variants are selected (10 *%* of each).

In rare variants analysis, we are interested in identifying regions with significant effects on the phenotype corresponding to the following set of hypotheses for each region

$$H_{0}: \forall j ~\gamma_{j}=0 \quad \text{verses}\quad H_{1}: \exists j\, \text{s.t.} \gamma_{j}\neq 0. $$

To test these hypotheses, we calculated the likelihood ratio of the selected model based on CCRS to the Null model, which does not include genotype variants in the model.

We evaluated the performance of the CCRS method compared to four other commonly applied methods: Collapsing [8] denoted here as Col, CAST [3], SKAT-O [17] and sparse principal regression (SPC) [20]. The collapsing method generates a binary variable for each region to represent whether the minor allele is observed. It then tests the association between the traits level and the new binary variable $I_{\{\sum _{j} \mathbf {x}_{j}>0\}}$ through $ \mathbf {y}=\alpha +I_{\{ \sum _{j} \mathbf {x}_{j}>0\}} {\beta } + \boldsymbol {T} \boldsymbol {\theta }+ \boldsymbol {\epsilon }$ regression model. The CAST method sums over all variants in the region and leads to $ \mathbf {y}=\alpha + \left (\sum _{j} \mathbf {x}_{j}\right) {\beta } + \boldsymbol {T} \boldsymbol {\theta }+ \boldsymbol {\epsilon }$. SKAT-O is a score based test, $(\mathbf {y}- \hat {\boldsymbol {\mu }})^{T} P_{\rho }(\mathbf {y}-\hat {\boldsymbol {\mu }})$ when ***β***_***k***_ in (1) follows an arbitrary distribution with mean 0 and variance *τ* and pairwise correlation *ρ* between different *β*_*jk*_s. Here, $\hat {\boldsymbol {\mu }}$ is the predicted mean of **y** under *H*_0_, $P_{\rho } = \boldsymbol {X}_{k} R_{\rho } \boldsymbol {X}_{k}^{T}$ is an *n*×*n* kernel matrix, *R*_*ρ*_=(1−*ρ*)*I*+*ρ* **1** **1** ^*T*^ where **I** is an *p*×*p* compound symmetric matrix, and **1** ^*T*^=(1,…,1)^*T*^.

To examine the impact of significance level on the false and true discovery rates, we considered both *α*=0.01 and 0.05 and calculated false discovery rate (FDR) and true positive discovery rate (TPR) defined as

$$\begin{array}{@{}rcl@{}} FDR&=&\mathrm{E} \!\left[F/R \mid R > 0\right] P\left[R>0\right],\\ TPR&=&\mathrm{E} \!\left[T/(M-R) \mid (M-R) > 0\right] P\left[(M-R)>0\right], \end{array} $$

where *F* is the number of false positives; *T* is the number of true discoveries; *R* is the total number of significant regions; and *M* is the total number of regions.

To select the best model based on the CCRS method, we set *ν* = 0.01 and *κ* = 0.5 after calculating BIC of the model over for different values of *ν* in {0.001,0.005,0.01,0.02,0.05}. Here, BIC of the model is defined as

$${} \text{BIC}(\nu)= \text{log}\{ \parallel \mathrm{y} - \mathbf{Z} \hat{\boldsymbol{\gamma}}(\nu) - \mathbf{T}\hat{\boldsymbol{\theta}}(\nu) {\parallel_{2}^{2}}/n \} +\text{log}(n)d(\nu)/n $$

where *d*(*ν*) is the number of effective parameters, $\hat {\boldsymbol {\gamma }}$ and $\hat {\boldsymbol {\theta }}$ minimize (2.4) with a given *ν* [34]. A larger penalty parameter *ν* might be applied for problems with larger number of variants in each region.

**Fig. 2:**
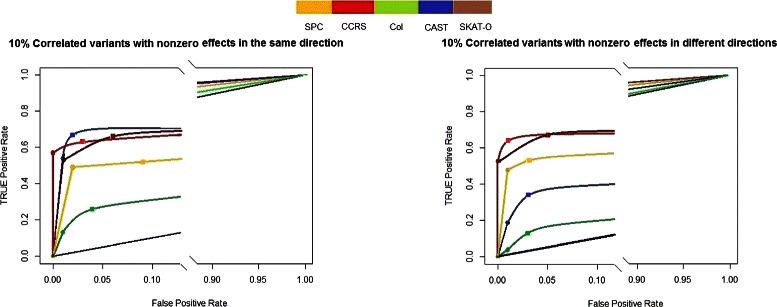
FDR vs. TPR of *Model-1* in left panel, and *Model-2* in right panel, for *α*=0.01(∙) and *α*=0.05(■), compare the CCRS performance with commonly applied methods, SPC, Col, CAST, SKAT-O

The results of the simulation study for *Model-1* and *Model-2* are shown in Fig. 2. The Col method does not have sufficient power to detect the associated regions. The CAST method shows better performance at the level *α*=0.05, when the direction of effects are the same. At the level *α*=0.05, the CCRS method shows better performance than SKAT-O when the direction of effects are different. At the level *α*=0.01, the CCRS and SKAT-O show nearly the same performance. It is clear from the figure that the CCRS method improves performance over the SPC method.

**Fig. 3:**
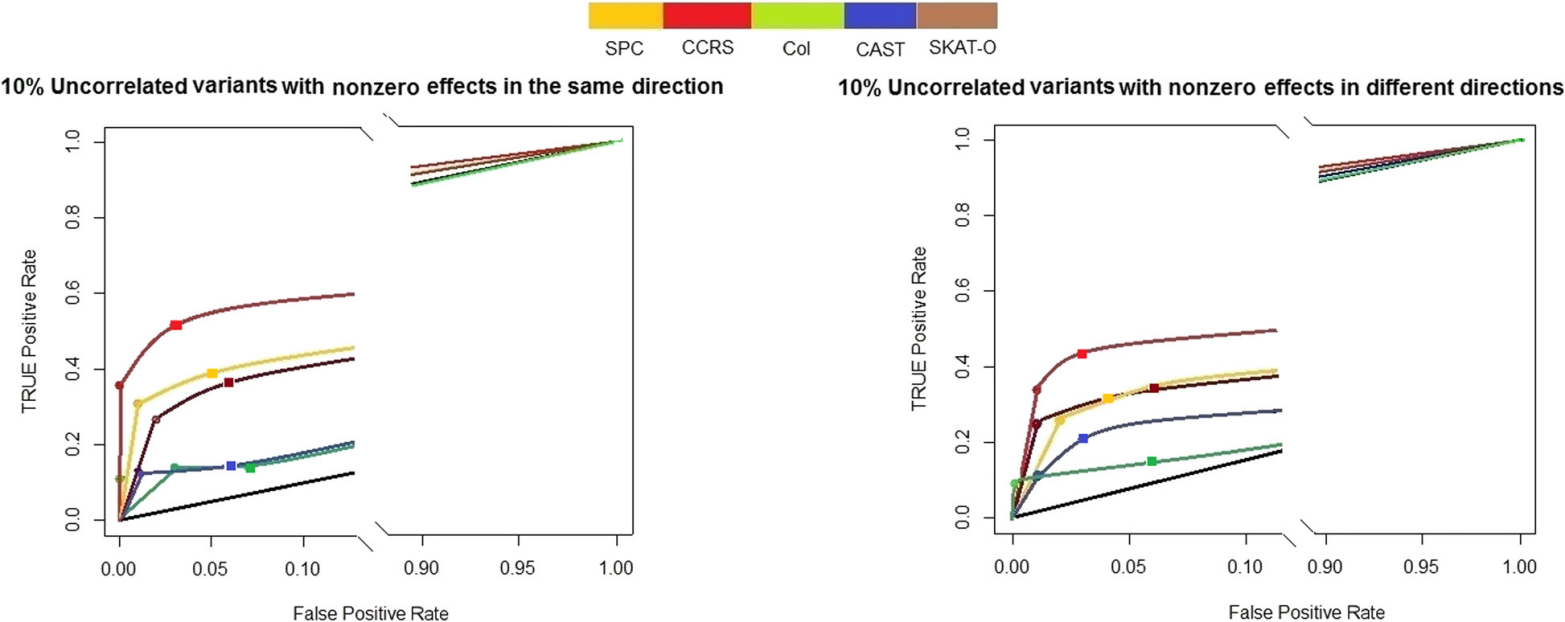
FDR vs. TPR of *Model-3* in left panel, and *Model-4* in right panel, for *α*=0.01(∙) and *α*=0.05(■), compare the CCRS performance with commonly applied methods, SPC, Col, CAST, SKAT-O

Figure 3 shows the result of simulation analysis for *Model-3* and *Model-4*. The CAST method for *Model-3* and the Col method for both *Model-3* and *Model-4* show poor performance. In both models, the CCRS shows noticeably better performance in both *α* levels. The influential regions in *Model-1* through *Model-4* have the same effect sizes on the phenotype. Hence, comparing Figs. 2 and 3 provides insight into understanding the influence of LD between causal variants and neutral variants on the power and accuracy of selection. The FDR of the Col and CAST methods shows the largest differences between these two figures. The FDR of CCRS is robust to the correlation among causal and neutral variants in comparison to the other methods.

**Fig. 4:**
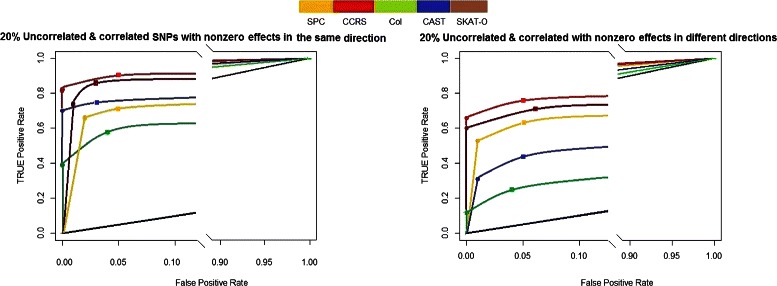
FDR vs. TPR of *Model-5* in left panel, and *Model-6* in right panel, for *α*=0.01(∙) and *α*=0.05(■), compare the CCRS performance with commonly applied methods, SPC, Col, CAST, SKAT-O

Figure 4 shows the result of analysis of *Model-5* and *Model-6* which include both correlated and uncorrelated effective variants. SKAT-O shows smaller FDR at the level *α*=0.05 in the left panel and slightly smaller TPR than CCRS, although at level *α*=0.01 CCRS shows better performance in terms of FDR and TPR. When the directions of effects are different (*Model-6*), right panel, CCRS outperforms the other methods.

The result of this simulation study shows that the CCRS performs better and more robust than other methods under a variety of genetic architectures, and it is much more prominent when the causal variants are not correlated with neutral variants in the region. Neglecting the presence of LD leads to overestimation of the overall effect of the regions. Although this overestimation might increase the power of detecting a region with some small effects that are correlated with some neutral variants, it increases the risk of missing more promising regions in procedure of multiple comparison of hypotheses testing.

### Real data analysis

We analyzed sequencing data from the Atherosclerosis Risk in Communities (ARIC) study [35]. The data are described more fully in [19]. Briefly, 496 African-American individuals were whole genome sequenced at an average depth of 6.3-fold using an Illumine HiSeq 2000 and, after alignment, approximately 31 million high quality variants were called using SNPTools. We present here the result of an association analysis of rare and low frequency variants (MAF≤0.05) with log transformed Apolipoprotein A1 levels (ApoA1). ApoA1 is a component of high density lipoprotein (HDL), which is associated with reduced risk of coronary heart disease [36, 37]. The protein promotes lipid efflux, including cholesterol, from tissues to the liver for excretion [38].

The genotype data includes 949,986 rare variants that are mostly in noncoding regions in the genome [19]. Therefore, we used a sliding window approach to define physical proximity (window). There are approximately 38 thousand consecutive windows each including 50 rare variants and stepping 25 variants until the next window. Therefore, by design, the windows overlap and the results of consecutive windows are not independent. To detect associated regions potentially influencing plasma ApoA1 levels, we used SPC, SKAT, SKAT-O, CAST and Col methods in addition to the CCRS method introduced here.

**Fig. 5:**
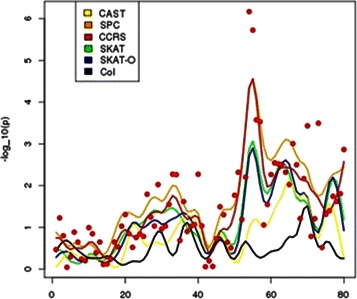
*p*-values of 80 windows around the selected window. The red points represent the calculated *p*-value by CCRS for the windows and each curves is smooth curve over calculated *p*-values by one approach; *yellow*: CAST test, *red*: CCRS, *green*: SKAT, *blue*: SKAT-O, *black*: Col, *orange*: SPC

To define the threshold for statistical significance taking into account multiple hypothesis testing, we ran 100,000 permutation test over 100 windows. Based on this threshold, 10^−6^, we detected one significantly associated region by the CCRS method. Figure 5 shows the *p*-values of 80 windows around this region. All of the approaches except CAST and Collapsing test have a peak in this region. The figure shows that the CCRS maintains power for detecting phenotype-influencing region while keeping the *p*-value of the null or neutral regions small. This is an important property of CCRS that controls the false discovery rate.

The region contains the gene, FAM78B, which is expressed at high levels in myocytes, fibroblasts, endothelial cells. Little is known about the function of FAM78B. However, within the promoter for FAM78B, three binding sites for the transcription factor PPARG and two binding sites for the transcription factor HNF1A have been identified (http://www.sabiosciences.com and [39]). Pi et al. [40] have shown a significant effect of PPARG on HDL and ApoA1; the major protein component of HDL [41]. PPARs are also expressed in the cardiovascular system such as endothelial cells, vascular smooth muscle cells and monocytes/macrophages (see for e.g. [42]).

## Discussion

We have introduced a new approach, CCRS, for the analysis of whole genome sequence data in order to identify regions of the genome (e.g. genes or other functional motifs) influencing a phenotype of interest. The CCRS improves the power of identifying a set of variants associated with a phenotype by taking into account the sparseness and LD structure in the data. The CCRS applies a concave penalized regression method after projecting the sequence variants in a full rank space that is more informative via sparse principal component analysis. By applying sparse PCA, the CCRS aims to enhance the information in the predictors instead of reducing dimension as typical application of sparse PCA, which might increase risk of missing important variants in rare variants analysis. While the first step of analysis (sparse PCA) is an unsupervised method, it does not increase the FDR of the method in the second step of the analysis.

Although the CCRS method can be applied to both common and rare variants, the focus of this analysis was on rare variants because of the role of these variants on phenotype variation. The CCRS method also can be easily expanded to logistic regression and applied for case control studies. However, we investigated the CCRS performance for quantitative traits while the overwhelming majority of the literature focusing on case/control studies and there is a daunting need to develop methods for quantitative traits.

Using simulated data, we show that the FDR of the CCRS method is smaller than other commonly applied genomic region-based test methods while it has higher power of identification in most of the situations. Furthermore, the FDR of CCRS is smaller and robust to the LD structure in the region in comparison to the other methods. While the statistical test for rare variants are typically region-based test, there is risk of overestimation of overall effect of regions by neglecting the LD between causal and neutral variants in the region. Consequently, the risk of missing promising regions might increase through multiple hypotheses testing.

Penalized regression and other shrinkage methods that have been introduced for sparse data applications can correctly select nonzero coefficients under specific conditions [43, 44]. Applying these approaches to large-scale genome sequence applications that include correlated variants due to LD might not lead to a true set of selected variants with nonzero coefficients. Addressing this challenge is difficult in rare variant analyses because each individual variant by itself includes little information. To resolve this problem, the CCRS reparameterizes the model via PCA restricted with *L*_1_ norm constraints to provide a full rank design matrix. Imposing *L*_1_ penalization in PCA generates a sparse loading matrix that renders the analysis interpretable. The CCRS method efficiently incorporates information from low frequency variants by generating new predictors that are much more informative. The CCRS uses a concave penalized regression model to simultaneously select the most important PCs regarding their association with the phenotype of interest, but also to estimate their effect sizes. The zero effect sizes can be uniquely identified due to the use of full rank approximation of the design matrix. The advantage of the concave penalty term is that the rate of shrinkage gets smaller as the effect size increases. In other words, the CCRS not only has the property of parsimony, it also avoids shrinkage over large effect sizes. Thus, the CCRS maintains power for detecting phenotype-influencing regions while keeping the *p*-value of the neutral regions small.

As an example real data application, we used the CCRS method and genome sequence data to analyze plasma ApoA1 levels, and one region met the experiment-wise criterion for statistical significance. The region contains the gene, FAM78B, which is expressed at high levels in myocytes, fibroblasts, endothelial cells (http://www.proteinatlas.org/ENSG00000188859-FAM78B/tissue). In a real application, annotation of the non-coding regions should be integrated into the analysis, and replication in an independent sample would be the next step to consider it as a novel discovery.

## Conclusions

Large-scale whole genome sequencing and high-powered computing are becoming more readily available and affordable. There is an emerging shift from sequencing and computing technologies toward study design, data processing algorithms, and statistical and informatics methods for extracting usable information from the very large amount of genome sequence data that are imminent. The CCRS method presented here for the first time is a practical, powerful and efficient method for taking into account the nature of whole genome sequence variation to identify regions of the genome influencing common complex risk factor phenotypes and diseases.
